# Integrated optical modulator manipulating the polarization and rotation handedness of Orbital Angular Momentum states

**DOI:** 10.1038/s41598-017-04118-5

**Published:** 2017-06-19

**Authors:** S. Faezeh Mousavi, Rahman Nouroozi, Giuseppe Vallone, Paolo Villoresi

**Affiliations:** 1 Department of Physics, Institute for Advanced Studies in Basic Sciences, 45195-1159 Zanjan, Iran; 2 Dipartimento di Ingegneria dell’Informazione, Università di Padova, I-35131 Padova, Italy

## Abstract

Recent studies demonstrated that the optical channels encoded by Orbital Angular Momentum (*OAM*) are capable candidates for improving the next generation of communication systems. *OAM* states can enhance the capacity and security of high-dimensional communication channels in both classical and quantum regimes based on optical fibre and free space. Hence, fast and precise control of the beams encoded by *OAM* can provide their commercial applications in the compatible communication networks. Integrated optical devices are good miniaturized options to perform this issue. This paper proposes a numerically verified integrated high-frequency electro-optical modulator for manipulation of the guided modes encoded in both *OAM* and polarization states. The proposed modulator is designed as an electro-optically active Lithium Niobate (*LN*) core photonic wire with silica as its cladding in a *LN* on Insulator (*LNOI*) configuration. It consists of two successive parts; a phase shifter to reverse the rotation handedness of the input *OAM* state and a polarization converter to change the horizontally polarized *OAM* state to the vertically polarized one. It is shown that all four possible output polarization-*OAM* encoded states can be achieved with only 6 *V* and 7 *V* applied voltages to the electrodes in the two parts of the modulator.

## Introduction

In the past decades, interests in exploiting Orbital Angular Momentum (*OAM*) as a new degree of freedom for encoding the information in optical communication channels have been enhanced^1^. Utilizing this technique in classical communication based on both optical fibre^2^ and free space^3–7^, and also in quantum communication^8–10^ demonstrated a promising increased data transmission rate for the future networks. In addition, secure transmission is one of the concerns of communication systems. Besides the classical cryptography techniques, Quantum Cryptography (*QC*) improved the security between authorized partners connected by a quantum channel. Quantum Key Distribution (*QKD*) protocols are used intensively for approaches of *QC*. As an example, *BB*84 *QKD* protocol employs four states belonging to two conjugate bases (such as horizontal, vertical, left circular and right circular polarization states) for data encoding^11, 12^. By combining polarization with *OAM*, generation of a rotation invariant qubit is possible. In this case, *QKD* is proved to be independent of alignment^13, 14^. This toolbox can be a good choice for free-space optical and quantum communication with moving objects, such as satellites or flying platforms. The rotation invariant qubit in *QKD* has been operated experimentally over a distance of 210 *m* ^15^. Moreover, the possibility of using *OAM* together with polarization for encoding the high-dimensional QKD states improves noise resistance and increase the content of information carried by each photon^16^.

Carrier modes with phase term of *exp*(*ilϕ*) have *OAM* corresponding to *l*ℏ; per photon. In this term *l* (*l* = 0, ±1, ±2, …) is the topological charge of the mode and its sign determines the rotation handedness of an *OAM* state. This mode can be polarized in any polarization state^17^.

High speed and high-frequency switching and modulation of a conventional communication optical channel can be achieved by tunable liquid crystal retarders^18, 19^ or via electro-optical effects in active integrated optical-based devices^20^. In the integrated optical devices, phase, amplitude, and polarization of the electric field of the optical communication signal are modulated using miniaturized waveguides with straight or interferometric configurations^21^. As it is mentioned above, one of the possible approaches to increase the capacity of an optical^7, 22–24^ or quantum^9^ channel is the using of an optical signal carrying *OAM*. Although integrated optically generation, detection, multiplexing, and switch of the optical signals with different *OAM* values are demonstrated^25–32^, but neither modulators nor switches for manipulating a specific guided mode carrying *OAM* integrated optically have been proposed. These modulators may have many applications in *OAM* based telecommunication in classical and quantum regimes.

**Figure 1:**
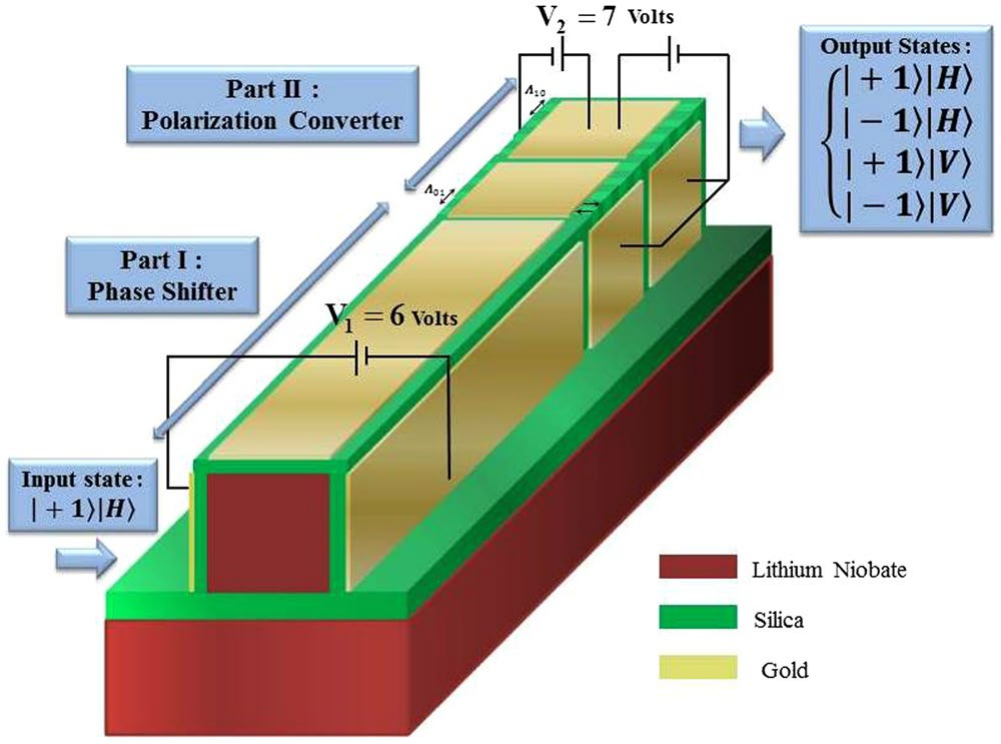
Schematic diagram of the proposed modulator with its input and output states.

In this paper, a modulator enabling control of the beams encoded by polarization-*OAM* states of |±1〉|*H*〉 is proposed for the first time to the best of our knowledge. The output states of the modulator are the four states representing a basis for the full four-dimensional polarization-OAM space. The *OAM* states (|*l* = −1〉 and |*l* = 1〉) and polarization states (|*H*〉 and |*V*〉) are considered as two encoding bases. In the proposed modulator, |+1〉|*H*〉 is assumed to be as the input state and four desired output quantum states of |−1〉|*H*〉, |−1〉|*V*〉, |+1〉|*V*〉, and |+1〉|*H*〉 can be achieved exploiting the advantages of a high-frequency electro-optically active Lithium Niobate on insulator (silica) photonic wire. Conversion of |+1〉|*H*〉 ⇔ |−1〉|*H*〉 can be obtained via rotation handedness reversal using a phase shifter part. Whereas, the conversion of |±1〉|*H*〉 ⇔ |±1〉|*V*〉 can be achieved in the polarization converter part. Figure 1 displays a schematic diagram of input state, the proposed modulator consists of two parts and its four desired output states.

## Results

### Structure of the Proposed Modulator

One of the waves carrying *OAM* is Laguerre-Gaussian (*LG*) optical mode^33^. In this section, an integrated optical modulator to manipulate the rotation handedness and polarization of the input |*LG*_+1,0_〉|*H*〉 (≡|+1〉|*H*〉) state is proposed and its different parts are explained in details. As it is shown in equation (1) and equation (2), the |*LG*_±1,0_〉 states can be built up with two Hermite-Gaussian (|*HG*_1,0_〉 and |*HG*_0,1_〉) modes with the relative phase differences of ±*π*/2^34^ (See Supplementary Information).

1 $$|L{G}_{1,0}\rangle=\frac{1}{\sqrt{2}}(|H{G}_{1,0}\rangle+i|H{G}_{0,1}\rangle),$$

2 $$|L{G}_{-\mathrm{1,0}}\rangle =\frac{1}{\sqrt{2}}(|H{G}_{\mathrm{1,0}}\rangle -i|H{G}_{\mathrm{0,1}}\rangle ).$$

**Figure 2:**
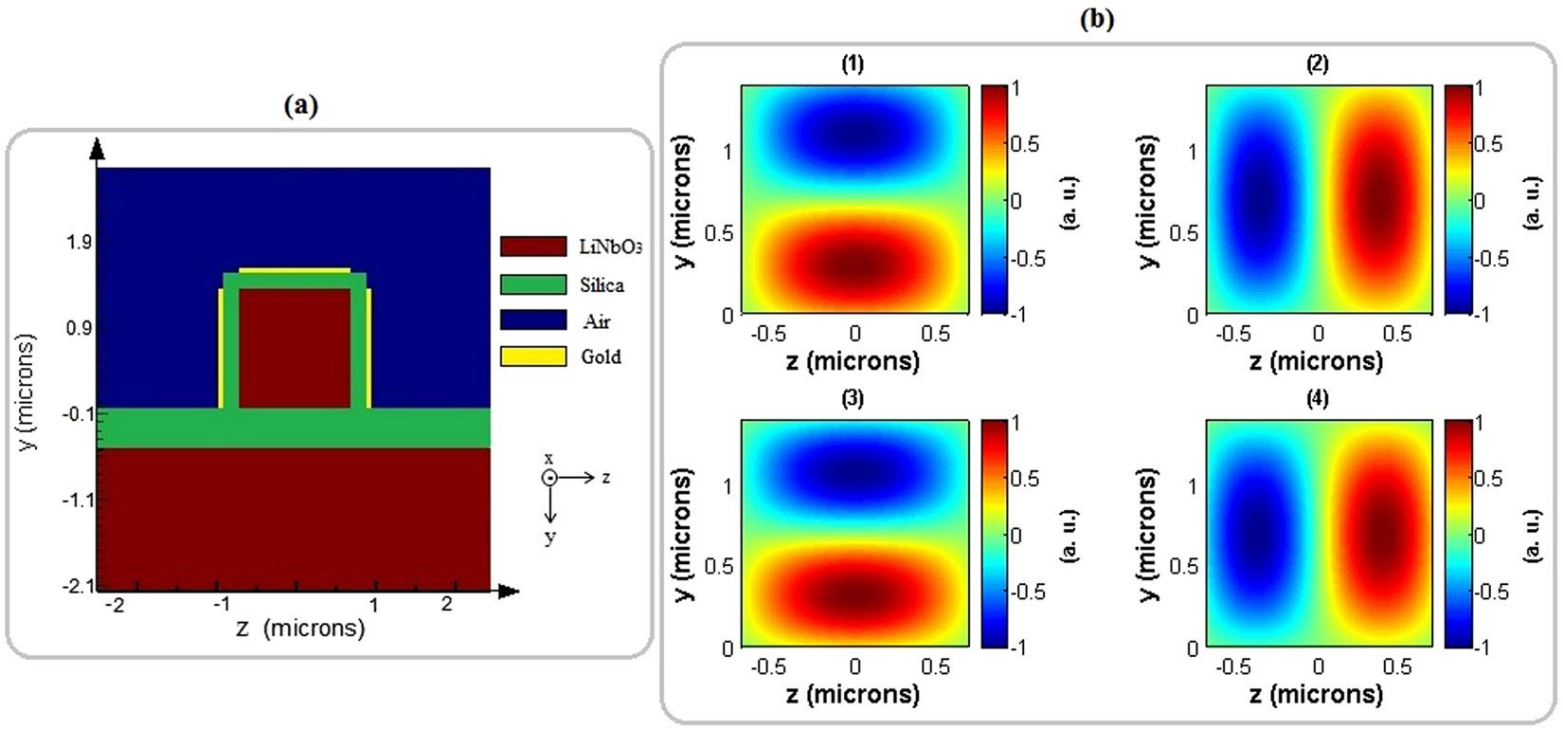
(**a**) Schematic representation of designed modulator. Red: *LiNbO*_3_, green: silica, yellow: gold electrodes and blue: air; (**b**) Normalized electric field distributions of the guided |*TE*_01_〉 (1), |*TE*_10_〉 (2), |*TM*_01_〉 (3), and |*TM*_10_〉 (4) modes in the proposed modulator.

In order to excite a less perturbed guided |*LG*_±1,0_〉, a symmetric waveguide in both horizontal and vertical directions is needed. Thus, designed modulator is assumed to be constructed of a rectangular shaped photonic wire. The ridge type *Y*_*cut*_-*LN* (*LiNbO*_3_) is used as the core of the designed photonic wire in order to manipulate the input state using electro-optic effect with appropriately designed electrodes in the lateral and top of it. Figure 2a illustrates the cross section of the 1.4 × 1.4 *μm* ^2^ sized photonic wire. A thin buffer layer of silica with the thickness of 200 *nm* operates as the top and lateral claddings, and a 500 *nm* thick layer plays the role of bottom cladding.

The calculated effective indices listed in Table 1 are obtained from the mode solution package of Lumerical software^35^ for the proposed modulator with mentioned dimensions. The results that have been gained for waves of 850 *nm* wavelength indicate that |*TE*_01_〉, |*TE*_10_〉, |*TM*_01_〉 and |*TM*_10_〉 modes which are needed for the composition of |*LG*_±1,0_〉 can be guided in the photonic wire. Figure 2b shows the calculated normalized electric field distributions of the guided modes inside the photonic wire.

**Table 1:** Calculated effective indices of the guided modes in the photonic wire

mode	effective index
|*TE*_01_〉	2.083887
|*TE*_10_〉	2.080837
|*TM*_01_〉	2.156396
|*TM*_10_〉	2.162771

As it is explained briefly in the introduction, proposed modulator should convert the input |+1〉|*H*〉 to one of the output states of |−1〉|*H*〉, |−1〉|*V*〉, |+1〉|*V*〉, and |+1〉|*H*〉. Therefore, the whole proposed modulator consists of a phase shifter followed by a polarization converter. In order to convert input |+1〉|*H*〉 to output |−1〉|*H*〉, the rotation handedness of |*LG*_1,0_〉 mode should be reversed. That means *l* index has to be changed from 1 to −1. Consequently, a *π* phase shift between guided |*TE*_10_〉 (|*HG*_10_〉) and |*TE*_01_〉 (|*HG*_01_〉) must be imposed in the first part of the modulator. Now, to achieve one of the |+1〉|*V*〉 and |−1〉|*V*〉 states from |±1〉|*H*〉 as the output state of the phase shifter part, the |*TE*_01_〉 (|*HG*_01_〉|*H*〉) and |*TE*_10_〉 (|*HG*_10_〉|*H*〉) should be converted to |*TM*_01_〉 (|*HG*_01_〉|*V*〉) and |*TM*_10_〉 (|*HG*_10_〉|*V*〉), simultaneously. In the other words, the first part operates as conversion of |*l* = +1〉|*H*〉 ⇔ |*l* = −1〉|*H*〉, and the second part operates as polarization conversion of |*l* = −1〉|*H*〉 ⇔ |*l* = −1〉|*V*〉.

In both parts of the proposed modulator which are introduced above, the electro-optic effect in *LN* is needed. Therefore, as demonstrated schematically in Fig. 2a, three gold-coated electrodes located surrounding the silica cladding is required to induce desired phase and refractive index changes in the phase shifter and the polarization converter parts, respectively. The location of electrodes is chosen in such a way that desired external electric fields can be applied in both parts. More details are described in the following of this section.

#### Part One: Phase Shifter

In order to convert input |*LG*_10_〉 mode to one of the output states with its reversed topological charge (|*LG*_−10_〉), one needs to impose *π* phase shift between two decomposed *HG* modes. Since two *HG* modes propagate together in an electro-optically active waveguide; hence any relative phase change can be induced by applying external electric field. In this way, effective refractive indices of two *HG* modes can be modified in such a way that the appropriate relative phase difference (*π*) is achieved. The input *LG* mode assumed to have *TE* (Horizontal, |*H*〉) polarization. Therefore two decomposed *HG* modes also have the same states of polarization. In order to impose *π* phase shift between two |*HG*_01_〉 and |*HG*_10_〉 modes, the external electric field must be applied in the *z* direction. Accordingly, their extraordinary effective refractive indices are changed as follows^21, 36^ (see Supplementary Information):

3 $${\rm{\Delta }}{n}_{e01}\cong -\frac{1}{2}{n}_{e01}^{3}{r}_{33}\langle{E}_{z}\rangle,$$

4 $${\rm{\Delta }}{n}_{e10}\cong -\frac{1}{2}{n}_{e10}^{3}{r}_{33}\langle {E}_{z}\rangle,$$

where Δ*n*_01_ and Δ*n*_10_ are deviation of effective refractive indices from their initial values (*n*_01_, *n*_10_) respectively when *E*_*z*_ as the external electric field in the *z* direction is applied and *r*_33_ is related electro-optic coefficient. As it is shown in equation (5), the total relative phase difference (Δ*ϕ*_*total*_) between |*HG*_01_〉 and |*HG*_10_〉 modes with wavelength (*λ*) when the phase shifter part has the length of *X* meter is the summation of two contributions:

*I*: is due to propagation (Δ*ϕ*_*prop*_), and

*II*: is due to electro-optic effect (Δ*ϕ*_*e*−*o*_).

5 $$\begin{array}{c}{\rm{\Delta }}{\varphi }_{total}={\rm{\Delta }}{\varphi }_{prop}+{\rm{\Delta }}{\varphi }_{e-o}=\frac{2\pi }{\lambda }(({n}_{TE,01}-{n}_{TE,10})+({\rm{\Delta }}{n}_{e01}-{\rm{\Delta }}{n}_{e10}))X\end{array}.$$

It is important to mention that, the phase shifter acts as the rotation handedness converter when the external electric field is applied. That means in the absence of external electric field the precise length of the phase shifter has to be adjusted in such a way that the Δ*ϕ*_*prop*_ be equal to *nπ*/2 (*n* = 1, 5, 9, …). This length is calculated to be 39.295 *mm* by using the parameters listed in Table 1 which are inserted to the first term of the equation (5). Independent of the first term in the equation (5), the conversion of input |*LG*_10_〉 to |*LG*_−10_〉 can only be achieved if the Δ*ϕ*_*e*−*o*_ is equal to *π*.

**Figure 3:**
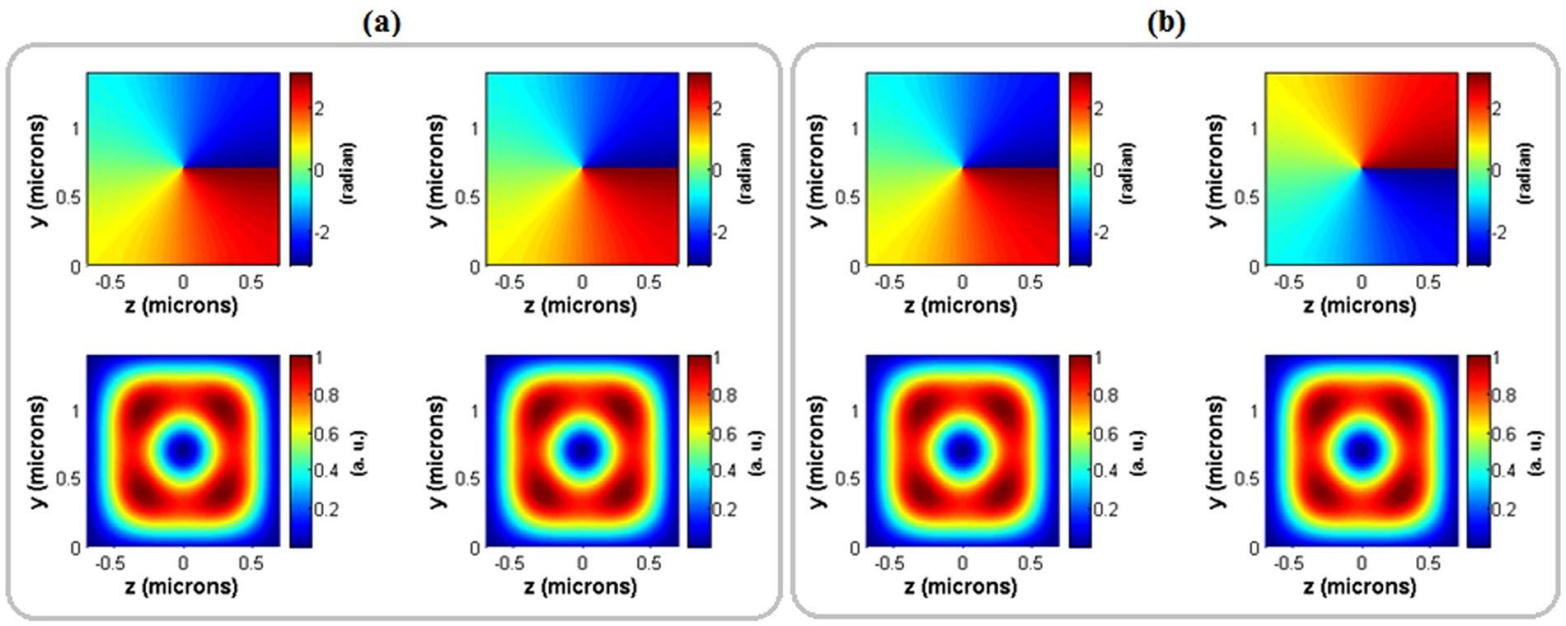
Left and right columns display the input and output phase patterns (top row) and power distributions (bottom row) of the horizontal polarized beam (|*H*〉) before (**a**) and after (**b**) the applied external electric field, respectively.

Results obtained from the calculations indicate that the conversion of rotation handedness can be achieved with only 6 *V* applied voltage between two lateral electrodes shown in Fig. 2a with ~1.5 *μm* distance and 39.158 *mm* length. The applied voltage of 6 *V* corresponds to ~4 × 10^6^ *V*/*m* applied external electric field which is much less than 22 *MV*/*m* as the coercive electric field for the bulk *LN*. Figure 3 illustrates the results obtained from the phase shifter when the external electric field is switched on (Fig. 3a) and switched off (Fig. 3b). The left and right columns in Fig. 3 demonstrate the phase patterns (top rows) and power distributions (bottom rows) of the input and output states, respectively. As Fig. 3a implies, the phase pattern and power distribution before and after phase shifter is conserved. This means that Δ*ϕ*_*prop*_ does not affect the input |*LG*_10_〉 state. Figure 3b shows that when the external electric field is switched on the power distribution conservation is valid during the handedness conversion of the input state |+1〉 to the output state of |−1〉. It means that the relative phase between two decomposed modes remains an odd integer multiple of *π*/2.

#### Part Two: Polarization Converter

As mentioned in previous part, the polarization of modes carrying OAM must be changed between two horizontal (|*H*〉) and vertical (|*V*〉) bases. This is possible when two decomposed |*TE*_01_〉 and |*TE*_10_〉 modes are converted to the |*TM*_01_〉 and |*TM*_10_〉 modes with orthogonal polarization. In other words, the conversion of |±*l*〉|*H*〉 ⇔ |±*l*〉|*V*〉 must be achieved. This can be provided via electro-optic effect in Periodically Poled Lithium Niobate (*PPLN*) waveguide^21^. Applying external electric field induces a perturbation in the dielectric constant tensor of the *LN*. Therefore as presented in the coupled mode equations (Eq. 6), two orthogonal mode bases can be converted to each other when an appropriate external electric field is applied^37^.

6 $$\begin{array}{ccc}\frac{d{A}_{1}}{dx} & = & -i\kappa {A}_{2}{e}^{i{\rm{\Delta }}\beta },\\ \frac{d{A}_{2}}{dx} & = & -i{\kappa }^{\ast }{A}_{1}{e}^{-i{\rm{\Delta }}\beta },\end{array}$$

where *A*_2_ and *A*_1_ are the amplitude of input *TE* (|*TE*_01_〉) or |*TE*_10_〉) and output *TM* (|*TM*_01_〉 or |*TM*_10_〉) modes, respectively and *κ* is the coupling coefficient between interacting modes. Δ*β* = (*β*_*TM*_ − *β*_*TE*_) − 2*π*/Λ represents the quasi-phase-matching (*QPM*) between two *TE* and *TM* modes with their propagation constants *β*_*TE*_ and *β*_*TM*_, respectively. 2*π*/Λ is the wave vector of the periodically poled domains with the wavelength of Λ. Equation (7) and equation (8) describe the dependency of the Λ to the effective refractive indices of two decomposed modes that have to be converted simultaneously (|*TE*_01_〉 ⇔ |*TM*_01_〉 and |*TE*_10_〉 ⇔ |*TM*_10_〉):

7 $${{\rm{\Lambda }}}_{01}=\frac{\lambda }{{n}_{o01}-{n}_{e01}},$$

8 $${{\rm{\Lambda }}}_{10}=\frac{\lambda }{{n}_{o10}-{n}_{e10}}.$$

The efficiency of these polarization conversions can be calculated using:

9 $${\eta }_{o}(x)={|\kappa |}^{2}{x}^{2}sin{c}^{2}(\sqrt{{|\kappa |}^{2}+{\rm{\Delta }}{\beta }^{2}}x),$$

where

10 $$|\kappa |=\frac{2}{\lambda }\frac{{{n}_{TM,eff}}^{2}{{n}_{TE,eff}}^{2}{r}_{51}|{E}_{y}|}{\sqrt{{n}_{TE,eff}{n}_{TM,eff}}}\,\sin (\pi D)\vartheta .$$

In this equation *D* as the duty cycle of the domain grating is assumed to be 0.5 and *r*_51_ as desired electro-optic coefficient of the *LN* is 28 × 10^−12^ *m*/*V*. *E*_*y*_ is the applied external electric field in the *y* direction and *ϑ* is the overlap integral between two interacting optical modes and the external electric field^38, 39^.

**Figure 4:**
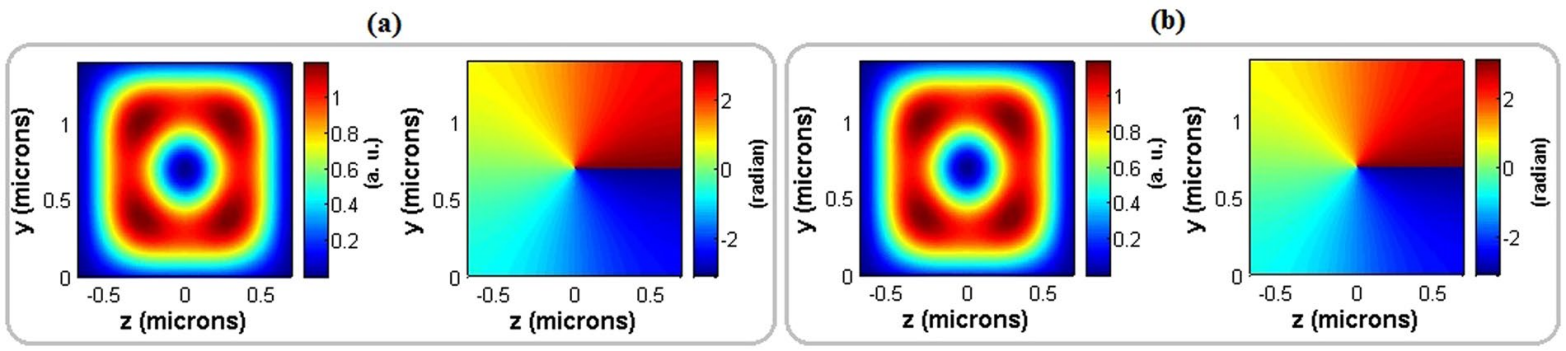
Power distribution (left) and phase pattern (right) of the input (**a**) and output (**b**) horizontally polarized optical modes in the polarization converter part of the modulator without applying external electric field.

Without applying external electric field, the phase of two input modes (|*TE*_01_〉 and |*TE*_10_〉) are only changed due to their propagation (see equation (5)). In order to conserve the rotation handedness of input *OAM* beam obtained from the phase shifter part of the modulator, the length of polarization converter part is calculated in such a way that the relative phase difference between two decomposed modes is an appropriate integer multiple of *π*/2. Therefore, the calculated total length of polarization converter part is 16.443 *mm*. Figure 4 illustrates the phase pattern and power distribution of the input and output horizontally polarized optical modes in the second part of the modulator when the external electric field is switched off. It implies that with precise control of the length of the polarization converter part, phase changes in the first part are not disturbed by second part of the modulator.

**Figure 5:**
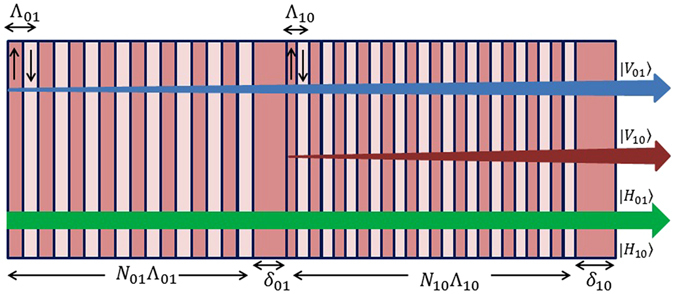
Schematic representation of polarization converter part (|*TE*_01_〉 ⇔ |*TM*_01_〉 and |*TE*_10_〉 ⇔ |*TM*_10_〉). Blue and red arrows display the productions of |*TM*_01_〉 and |*TM*_10_〉 from the first and second sections with domain wavelengths of Λ_01_ and Λ_10_, respectively. The green arrow shows that input |*TE*_01_〉 and |*TE*_10_〉 modes are not changed when external electric field is not applied.

Since the polarizations of two decomposed optical modes have to be converted independently, the polarization converter part should contain both domain wavelengths shown in equation (7) and equation (8). Therefore, polarization converter part is designed in such a way that first converts the |*TE*_01_〉 to |*TM*_01_〉 and then in a successive section converts the |*TE*_10_〉 to |*TM*_10_〉. Figure 5 displays the designed polarization converter part schematically. By applying external electric field, the first section that consists of *N*_01_ domain inversions with the wavelength of Λ_01_ provides the desired conversion of |*TE*_01_〉 ⇔ |*TM*_01_〉 while the other conversion (|*TE*_10_〉 ⇔ |*TM*_10_〉) is not achieved. On the other hand, in the second section with the wavelength of Λ_10_, the second desired conversion (|*TE*_10_〉 ⇔ |*TM*_10_〉) is dominant, while the output modes from 01 section (|*TM*_01_〉) are conserved.

**Figure 6:**
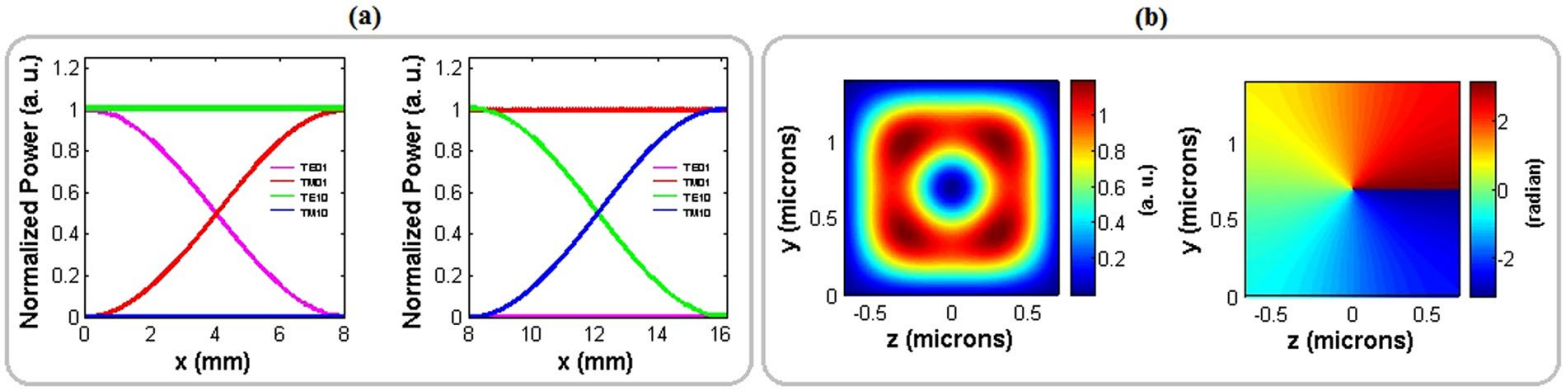
(**a**) Normalized power evolution of all interacting |*TE*_01_〉 (magenta), |*TM*_01_〉 (red), |*TE*_10_〉 (green) and |*TM*_10_〉 (blue) modes along the polarization converter part of the modulator for two successive sections with domain wavelengths Λ_01_ (left) and Λ_10_ (right); and (**b**) Power and phase of the emitted vertically polarized OAM carrying beams after applying external electric field.

Figure 6a shows the normalized power evolution of all interacting modes (|*TE*_01_〉, |*TM*_01_〉, |*TE*_10_〉 and |*TM*_10_〉) along the polarization converter part of the modulator. The length of both sections (*N*_01_Λ_01_ and *N*_10_Λ_10_) is 8.143 *mm*. With these propagation lengths for full polarization conversions, the applied voltage between the top and two lateral electrodes (see Fig. 1) is 7 *V*.

Although the polarization conversion is achieved when the external electric field is applied, but in principle additional phase deviations due to the propagation and electro-optically changes in refractive indices of converted and non-converted modes is mandatory^40^. These phase changes should be compensated in such a way that the relative phase difference between two decomposed and polarization converted modes of |*TM*_01_〉 and |*TM*_10_〉 is the desired integer multiple of *π*/2. This will guaranty the conservation of rotation handedness obtained from the first part of the modulator. The electro-optically induced phase changes in the both sections of the polarization converter (see Supplementary Information) are cancelled in the periodical domain inversion structure of PPLN^41^. But two additional propagation lengths *δ*_01_ and *δ*_10_ must be used after the two sections of polarization converter without domain inversion and the applied external electric field to compensate the relative phase changes owing to different propagation velocities. All of the parameters used in the polarization converter part of the modulator is summarized in Table 2.

**Table 2:** Characteristic data of designed PPLN-based polarization mode converter.

*N*_01_	Λ_01_ (*μm*)	*δ*_01_ (*μm*)	*N*_10_	Λ_10_ (*μm*)	*δ*_10_ (*μm*)
696	11.7	71.68	783	10.4	85.32

Result obtained from the polarization converter part of the modulator when the external electric field is switched on is illustrated in Fig. 6. *TM* polarized decomposed modes of |*TM*_01_〉 and |*TM*_10_〉 without any relative phase disturbance is achieved with an appropriate voltage in the engineered lengths of different sections in the polarization converter part. The phase pattern in Fig. 6b is the same as the phase pattern of the input optical mode shown in Fig. 4. This indicates no rotation handedness change during the propagation along the polarization converter part of the modulator.

## Discussion

This work proposes a numerically verified integrated optical modulator to be accomplished for controlling optical modes encoded on two *OAM* states (|*l* = −1〉 and |*l* = 1〉) and polarization states (|*H*〉 and |*V*〉). Proposed modulator is configured as a *Y*_*cut*_ *LN* on insulator (Silica) photonic wire to exploit the electro-optical effects for high-frequently manipulating the polarization and rotation handedness of polarization-*OAM* encoded optical states. The function of this modulator is defined in such a way that manipulates the input |+1〉|*H*〉 state and produces four desired states of |−1〉|*H*〉, |−1〉|*V*〉, |+1〉|*V*〉 and |+1〉|*H*〉. The designed modulator operates as a switch by applying appropriate external electric field to its two successive parts: phase shifter and polarization converter. If the applied voltage to the phase shifter (*V*_1_ = 6 *V*) is switched on, the rotation handedness of input state is reversed while its polarization state is conserved. In the other words, the conversion of |+1〉|*H*〉 ⇔ |−1〉|*H*〉 is achieved. Additionally, by switching the polarization converter’s voltage (*V*_2_ = 7 *V*) on, the horizontal polarization state of input mode is converted to vertical state while its rotation handedness is still maintained. The desired conversion of this part is |±1〉|*H*〉 ⇔ |±1〉|*V*〉. In both parts, switching the voltages off leads to prevention of mentioned conversions. The operation of proposed modulator is summarized in Table 3. These generated polarization-*OAM* encoded optical states can be used in classic and quantum high-dimensional communication and cryptography. It is worth noticing that for a complete exploitation in quantum key distribution applications, it will also be necessary to generate some superpositions between two four states that are listed in Table 3 and form a basis of the four-dimensional polarization-*OAM* space. The investigation of the generation of such superpositions by an integrated modulator is left for future studies.

**Table 3:** Designed switch for generating desired optical states ﻿when the input state of the modulator is﻿ |+1〉|H〉.

*V*_1_	*V*_2_	state
on	on	|−1〉|*V*〉
on	off	|−1〉|*H*〉
off	off	|+1〉|*H*〉
off	on	|+1〉|*V*〉

## Electronic supplementary material

Supplementary Information
