# Supplementary material for: Integrated optical modulator manipulating the polarization and rotation handedness of Orbital Angular Momentum states
Source: Sci Rep. 2017 Jun 19;7:3835. doi: 10.1038/s41598-017-04118-5 (PMC5476591; doi:10.1038/s41598-017-04118-5)
Supplement: Supplementary file 1 — Supplementary Information [file 41598_2017_4118_MOESM1_ESM.pdf]

Supplementary Information for:
Integrated optical modulator manipulating the polarization and rotation handedness of Orbital Angular Momentum states

S. Faezeh Mousavi, Rahman Nouroozi, Giuseppe Vallone, and Paolo Villoresi

Decomposition of OAM carrying modes

One of the waves carrying OAM is Laguerre-Gaussian (LG) optical mode which its electric field, $E_{l,p}$, is described in equation (S1). LG mode is a solution of the paraxial Helmholtz wave equation in the cylindrical coordinates (ρ, ϕ, z).

$$E_{l,p}(\rho, \phi, z, t) = A_{l,p}(\rho, \phi, z)e^{i\omega t} \tag{S1}$$

In this equation, ω is angular frequency and $A_{l,p}$ is the LG complex amplitude of order (l , p) defined as:

$$A_{l,p}(\rho, \phi, z) = A_0 \frac{w_0}{w(z)} (\frac{\rho}{w(z)})^{|l|} L_p^{|l|}(\frac{2\rho^2}{w^2(z)}) e^{-\frac{\rho^2}{w^2(z)}} e^{-i(k\frac{\rho^2}{2R(z)}+kz+l\phi-(|l|+2\phi+1)\zeta(z))} \tag{S2}$$

where k is the wave number of a wave that has Gaussian distribution with waist radius, width, and curvature of w_0, $w(z)$ and $\rho(z)$, respectively. Furthermore, A_0 is a normalization constant, $\zeta(z)$ determines Gouy phase shift and $L_p^{|l|}$ is defined as the generalized *Laguerre* polynomial function. $LG_{l,p}$ modes form a complete orthogonal basis.

One of the solutions to the paraxial Helmholtz wave equation in the Cartesian coordinates (x, y, z) is Hermite–Gaussian (HG) mode. Equation (S3) represents the complex amplitude of an optical HG mode [1].

$$A_{m,n}(x, y, z) = A_0 \frac{w_0}{w(z)} H_m(\frac{\sqrt{2}x}{w(z)}) H_n(\frac{\sqrt{2}y}{w(z)}) e^{-i(k\frac{x^2+y^2}{2R(z)}+kz-(m+n+1)\zeta(z))} \tag{S3}$$

In this equation, H_m determines the *Hermite* polynomial function of order m. Since the $HG_{m,n}$ modes form another complete orthogonal set of modes; therefore, any monochromatic optical mode can be understood as the superposition of $HG_{m,n}$ modes with the same optic axis, waist size, and waist position, but different mode indices, n and m. Thus, the $LG_{l,p}$ mode can also be decomposed in the bases of $HG_{m,n}$ modes as described in equation (S4) [2].

$$|LG_{l,p}\rangle = \sum_{n=0}^{\infty}\sum_{m=0}^{\infty} |HG_{n,m}\rangle\langle HG_{n,m}|LG_{l,p}\rangle \tag{S4}$$

In this way, decomposition of a $LG_{l,p}$ mode with l and p indices to the $HG_{n,m}$ modes with different orthonormal m and n indices can be understood through the equations:

$$l(n, m) = n - m, \qquad p(n, m) = min(n, m). \tag{S5}$$

As an example $[l = 1, p = 0]$ and $[l = -1, p = 0]$ are results of the pairs $[n = 1, m = 0]$ and $[n = 0, m = 1]$ with different relative phases $\pi/2$ and $-\pi/2$, respectively. Therefore they can be written as

$$|LG_{1,0}\rangle = \frac{1}{\sqrt{2}}(|HG_{1,0}\rangle + i|HG_{0,1}\rangle) \tag{S6}$$

$$|LG_{-1,0}\rangle = \frac{1}{\sqrt{2}}(|HG_{1,0}\rangle - i|HG_{0,1}\rangle) \tag{S7}$$

.

Perturbation solution of waveguide eigenproblem

The problem of various small imperfections and perturbations imposed on exact eigenmode of waveguide can be estimated via perturbation theory. By considering Maxwell's equations for definite-frequency (ω) fields that depend on time as $e^{-i\omega t}$, the hermitian generalized eigenproblem of waveguide can be described as:

$$\widehat{A}|\psi\rangle = \beta\widehat{B}|\psi\rangle \tag{S8}$$

In this equation, $\widehat{A}$ and $\widehat{B}$ are Hermitian operators defined by:

$$\widehat{A} \equiv \begin{bmatrix} \omega\varepsilon/c - \frac{c}{\omega}\overrightarrow{\nabla}_t \times \frac{1}{\mu}\overrightarrow{\nabla}_t\times & 0 \\ 0 & \omega\mu/c - \frac{c}{\omega}\overrightarrow{\nabla}_t \times \frac{1}{\varepsilon}\overrightarrow{\nabla}_t\times \end{bmatrix} \tag{S9}$$

$$\widehat{B} \equiv \begin{bmatrix} 0 & -\widehat{z}\times \\ \widehat{z}\times & 0 \end{bmatrix} = \begin{bmatrix} & & & 1 \\ & & -1 & \\ & -1 & & \\ 1 & & & \end{bmatrix} = \widehat{B}^{-1} \tag{S10}$$

and $|\psi\rangle$ is the electromagnetic field pattern as a function of z. For guided modes of a waveguide, the eigenvalues are a discrete sequence β_n with eigenstates $|n\rangle$.

Employing the perturbation theory formulations derived for quantum mechanics, the effects of a small perturbation ΔA are calculated. Therefore, the first correction to an eigenvalue β_n is given by [3]:

$$\delta\beta_n^{(1)} = \langle n|\Delta\widehat{A}|n\rangle \tag{S11}$$

One of the most general and important forms of waveguide perturbation is when refractive index, n, of a guided mode is changed by a small Δn. In electro optic effect, Δn is proportional to external electric field linearly as $\Delta n = -\frac{1}{2}n^3 r_{33}E_z$ for TE modes ($|n\rangle = |\psi_{TE}\rangle$) and as $\Delta n = -\frac{1}{2}n^3(r_{13}E_z + r_{22}E_y)$ for TM modes ($|n\rangle = |\psi_{TM}\rangle$). Accordingly, the perturbed refractive indices can be represented as:

$$n'_{TE} = n_{TE} - \frac{1}{2}n_{TE}^3 r_{33}\langle\psi_{TE}|E_z|\psi_{TE}\rangle \tag{S12}$$

$$n'_{TM} = n_{TM} - \frac{1}{2}n_{TM}^3(r_{13}\langle\psi_{TM}|E_z|\psi_{TM}\rangle + r_{22}\langle\psi_{TM}|E_y|\psi_{TM}\rangle) \tag{S13}$$

Implementation

For implementation of the modulator proposed in this paper, the procedures listed below are suggested [4, 5, 6, 7]:

1) Exploiting plasma enhanced chemical vapor deposition (PECVD) method for coating silica layer on Y_{cut} LN substrate (first layer). This silica layer would be as the bottom clad of the phonic wire.

2) A He^+ ion implanted Y_{cut} LN layer (second layer) can be used to bond to the insulator on LN layer (first layer) prepared in the first step.

3) Annealing the bonded layers to improve bonding strength and split thin layer of Y_{cut} LN along implanted ions. This layer that would be remained on the $SiO_2 - LN$, is the core of modulator.

4) Applying Chemical Mechanical Polishing (CMP) on the films to improve the surface roughnesses of resulted LN on Insulator (LNOI) wafer.

5) Using Plasma etching for slicing the LN part of resulted LNOI wafer and achieving a core with rectangle cross section attached to bottom silica clad and LN substrate.

6) Using photolithography for fabricating comb-like electrodes and then applying electric field to pole the etched LN (PPLN) core periodically. For forming $PPLN$ in this modulator, structure with two part, any one with its specific wavelength is recommended.

7) Exploiting PECVD method for coating thin layer of SiO_2, as top and lateral clads, on etched $LiNbO_3$ core; and then slicing the resulted coated wafer by plasma etching.

8) Applying PECVD method for coating thin layer of Au on SiO_2 layer (top and lateral clads), as top and lateral electrodes.

9) Using Plasma etching to form Au electrodes in the desired shapes.

References

[1] Saleh, B. E. A. & Teich, M. C. *Fundamentals of photonics; 2nd ed.* Wiley series in pure and applied optics (Wiley, New York, NY, 2007).

[2] O'Neil, A. T. & Courtial, J. Mode transformations in terms of the constituent hermite–gaussian or laguerre–gaussian modes and the variable-phase mode converter. *Optics communications* **181**, 35–45 (2000).

[3] Steven, G. Johnson *et al.* Low-loss asymptotically single-mode propagation in large-core omniguide fibers. *OPTICS EXPRESS* **9**, 748–779 (2001).

[4] Gui, L. *Periodically poled ridge waveguides and photonic wires in LiNbO3 for efficient nonlinear interactions / Li Gui.* Ph.D. thesis, Paderborn, Univ (2010).

[5] Hu, H, Ricken, R. & Sohler, W. Lithium niobate photonic wires. *OPTICS EXPRESS* **17**, 24261–24268 (2009).

[6] Rabiei, P, Ma, J., Khan, S., bibinfoauthorChiles, J., & Fathpour, S. Heterogeneous lithium niobate photonics on silicon substrates. *OPTICS EXPRESS* **21**, 25573–25581 (2013).

[7] Ueno, W. *et al.* Entangled photon generation in two-period quasi-phase-matched parametric down-conversion. *OPTICS EXPRESS* **20**, 5508–5517 (2012).
